# Education as a factor in social participation for people affected by disabilities in communities endemic to neglected tropical diseases in Benin and Côte d’Ivoire, West Africa

**DOI:** 10.3389/fresc.2026.1810112

**Published:** 2026-09-23

**Authors:** Fifamin Noël Christelle Gbaguidi, Florentine Adjouavi Houedenou, Ginette Victoire Senami Gandigbe, Sonagnon Ines Elvire Agbo, Yévèdo Borel Tossou, Sètondji Diane Edwige Zanvo, Karine Lucrèce Marie Codjo-Seignon, Sèdjro Gimatal Esaï Anagonou, Dagbédji Damien Toffa, Franck Zinsou Mignanwande, Paule Héléna Biaka, Mamadou Kaloga, Anna Gine-March, Manuel Pozzati, Roch Christian Johnson

**Affiliations:** 1 Centre Interfacultaire de Formation et de Recherche de Environnement Pour le Développement Durable, Université D’Abomey-Calavi, Abomey-Calavi, Bénin; 2 Ecole Doctorale des Sciences de la Santé de Cotonou, Université D’Abomey-Calavi, Cotonou, Bénin; 3 Institut des Sciences Anthropologiques de Développement, Université Félix Houphouet Boigny de Cocody, Abidjan, Côte d'Ivoire; 4 Programme national de lutte contre l'ulcère de Buruli et les maladies cutanées ulcéreuses endémiques, Abidjan, Côte d'Ivoire; 5 Anesvad Fondation, Bilbao, Spain; 6 Fondation Raoul Follereau, Paris, France

**Keywords:** Benin, Côte d'Ivoire, disability, education, social participation

## Abstract

**Introduction:**

Disability significantly affects access to fundamental rights, including education. This study aimed to analyse the factors associated with non-schooling among people with disabilities due to Neglected Tropical Diseases and other causes in Benin and Côte d’Ivoire.

**Methods:**

This was a descriptive cross-sectional study with an analytical focus using a sequential mixed-methods formative approach. Quantitative data were collected from 841 people with disabilities using structured questionnaires and analyzed with SPSS version 25 to identify factors associated with non-schooling. Qualitative interviews were thematically analyzed to explore participants’ experiences and needs, which informed the co-development of three context-adapted intervention modules. The modules were subsequently piloted and qualitatively evaluated among a small group of participants.

**Results:**

Factors associated with non-schooling included multiple disabilities [ORa = 3.91; 95% CI (2.01–7.2); *p* < 0.001], sensory disability [ORa = 1.47; 95% CI (1.00–2.16); *p* = 0.048], restrictions on social participation [ORa = 1.63; 95% CI (1.19–2.23); *p* = 0.002] and country of origin [ORa = 3.10; 95% CI (2.28–4.23); *p* < 0.001]. The pilot qualitative evaluation suggested perceived improvements in self-esteem, social participation, and engagement in income-generating activities among intervention participants.

**Conclusion:**

These findings highlight the importance of addressing educational barriers faced by people with disabilities. They also suggest that context-adapted educational and psychosocial interventions may support social participation, although further studies are required to evaluate their effectiveness.

## 1 Introduction

Disability significantly affects access to fundamental rights, including education. The World Health Organization estimated that 1.3 billion people are experiencing significant disability, which represents 16% of the global population (1). In Africa, the percentage ranges from 2%–5%, but in some countries, it is probably closer to 20%–22% owing to the prevalence of conflict, forced displacement, and lack of access to adequate medical services (2). There are other causes, such as chronic diseases and neglected tropical diseases (NTDs) (1, 3, 4). The consequences are manifold: health, economic, and social. Among them is education.

Education is an important determinant of health and social inclusion. However, people with disabilities often experience significant barriers to accessing education, including stigma, lack of inclusive infrastructure, and limited policy implementation. A positive correlation between education and health has already been established (5). Education is therefore essential for ensuring overall physical, mental, and social well-being (6).

Evidence from African countries highlights that barriers to education among people with disabilities are widespread across the continent. Studies conducted in many African countries have documented similar challenges such as stigma, inadequate inclusive education systems, and structural inequalities that limit access to schooling for people with disabilities (7–9). These findings emphasize that despite variations in national contexts, common patterns persist across sub-Saharan Africa, particularly in rural and underserved settings where health conditions such as the NTD further exacerbate vulnerability. A previous study conducted in Benin and Côte d’Ivoire showed that people with disabilities who have completed at least primary school face fewer barriers to social participation than those who have never attended school, and that as the level of education increases, restrictions on social participation decrease (10). Accordingly, this study was designed as a sequential mixed-methods formative study. The first phase aimed to identify factors associated with non-schooling among people with disabilities living in NTD-endemic communities in Benin and Côte d’Ivoire. The second phase explored participants’ experiences and needs through qualitative inquiry. The findings from these two phases informed the co-development of context-adapted educational intervention modules, which were subsequently piloted and qualitatively evaluated. Together, these complementary phases sought to generate evidence for strengthening educational inclusion and promoting social participation among people with disabilities.

## 2 Materials and methods

### 2.1 Study design and settings

This was a descriptive cross-sectional study with an analytical focus using a sequential mixed-methods formative approach (quantitative and qualitative) conducted in Benin (Ouidah, Lalo, Allada, Pobè) and Côte d’Ivoire (Divo, Yamoussoukro, Tiassalé) (Figure 1). These localities were selected because of their endemicity for NTDs. The study was conducted from December 2021 to June 2023.

**Figure 1:**
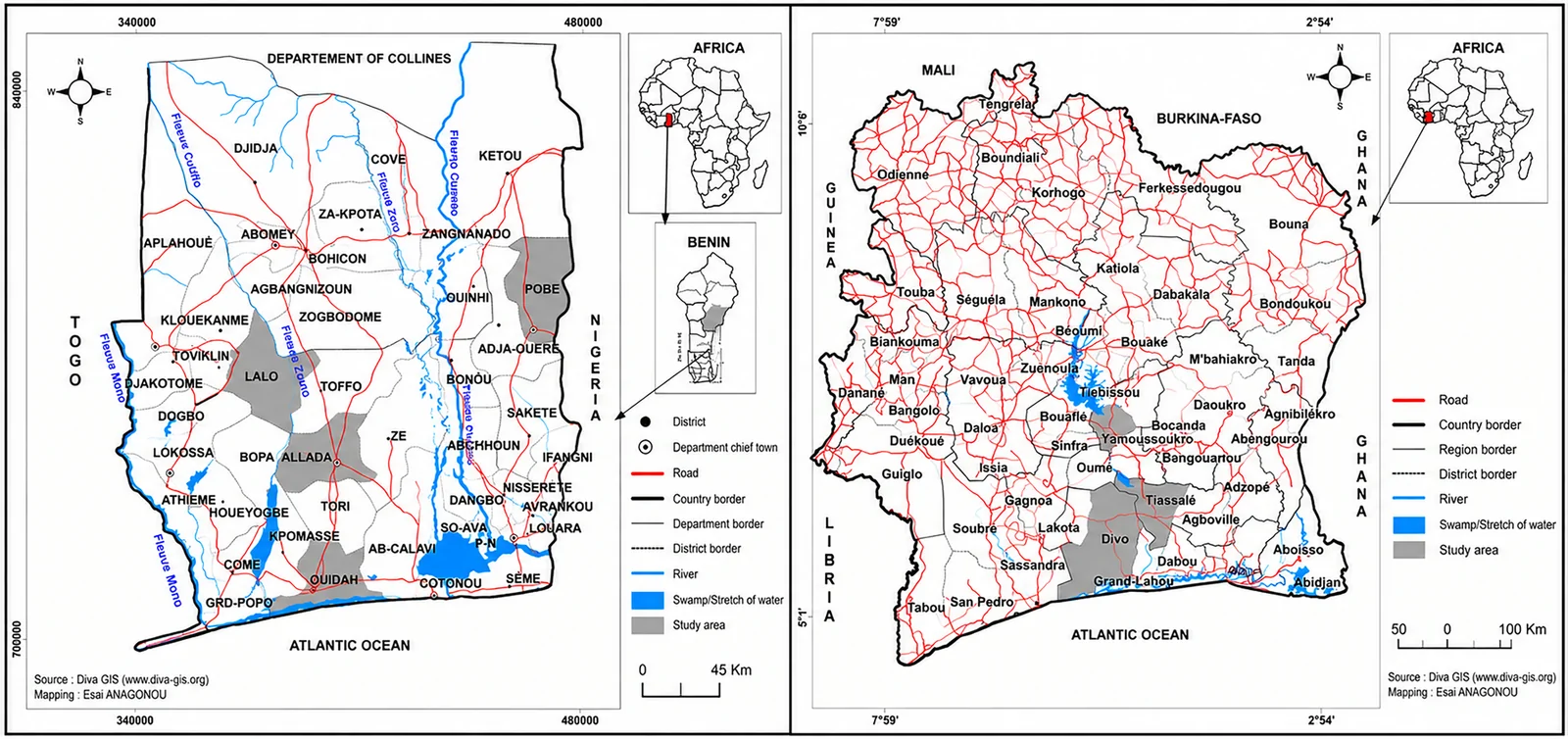
Maps of the study areas in côte d’Ivoire (left) and Benin (right). (Left) reproduced from “Map showing study sites in Côte d'Ivoire” (right) reproduced from “Map showing study sites in Benin” by Tossou et al, licensed under CC BY 4.0.

### 2.2 Ethical aspects

This study was conducted as part of a larger research project on disability and social participation in Benin and Côte d’Ivoire. Ethical approval was obtained from the ethics committee of the University of Parakou (CLERB-UP) in Benin under authorization N°0492/CLERB-UP/P/SP/R/SA of 19/11/2021 and by the National Ethics Committee for Life Sciences and Health (CNESVS) of Côte d’Ivoire under authorization N/Ref: 076-22/MSHPCMU/CNESVS- kp of 28/07/2022. All participants provided informed consent. Written informed consent was obtained from the participants and/or minors’ legal guardian/next of kin for the publication of any potentially identifiable images or data included in this article. It was made clear that there would be no consequences for those who chose not to participate in the study. In addition, all the data collected were anonymized to preserve the identity of the participants.

### 2.3 Target population

This study included people with disabilities due to NTDs and other causes. Participants were identified using health center database and community networks. The inclusion criteria were individuals living with a disability, regardless of age, sex, or cause. Family members (ascendants or guardians), caregivers (social workers, nurses, doctors and physiotherapists) and opinion leaders (village chiefs, religious authorities and traditional practitioners) who provided consent were also included.

### 2.4 Sample size and sampling methods

#### 2.4.1 People with disabilities

The target sample size was 841 people with disabilities (418 in Benin and 423 in Côte d’Ivoire), including both children and adults.

Schwartz's formula was used to determine the sample size in each country. A review of the literature revealed limited recent data on disabilities associated with neglected tropical diseases. In the absence of reliable prevalence estimates, a prevalence of 0.5 was assumed. The application of Schwartz's formula in this study was as follows:

$$n=(Z\alpha^{2}pq)/i^{2}$$

*n* = sample size; i = 5% (desired precision); *p* = 50%; q = 1-*p* = 50; Z*α* = 1.96 (value of the reduced deviation at a risk equal to 5%); Hence *n* = 384.

Thus, the minimum sample size obtained is 384. Assuming an acceptable margin of error of 5%, the sample size was 403 in each target country (806 participants for both). Given the challenges associated with field conditions, a snowball sampling approach was adopted to find the participants.

#### 2.4.2 Other population

Caregivers, district chiefs and religious leaders were selected on the basis of the locations of the communities in which people with disabilities belong. Moreover, the family and community members of people with disabilities who received the training were included. Their consent was also obtained.

### 2.5 Variables

To identify factors associated with non-schooling among people with disabilities, the following variables were measured:

- **Sociodemographic:** age, sex, and education level;
- **Clinical:** type of disability and cause of disability;
- **Social:** restrictions on social participation (social participation and restrictions were measured using the context-specific World Health Organization Participation Scale (10).

### 2.6 Data collection tools

Standardized questionnaires were developed on the basis of the literature and adapted to the local context to collect the data.

### 2.7 Data processing and analysis

Using the KoboCollect tool, quantitative data were collected from 841 people with disabilities. These data were then exported to Excel to ensure completeness and accuracy. Data analysis was carried out via the Statistical Package for Social Science (SPSS version 25). Quantitative variables are expressed as the means and standard deviations, whereas qualitative variables are summarized as frequencies. Means were compared via Student's t test, and frequencies were compared via Pearson's chi-square test or Fisher's exact test, as appropriate. A bivariate analysis using logistic regression was performed, and variables with a *p* value of less than 20% (*p* = 0.2) were retained for multivariate analysis. Multivariate analysis was performed via an iterative stepwise logistic regression model (Wald top-down). For all comparisons, a *p* value of less than 5% (*p* = 0.05) was considered to indicate statistical significance.

All interviews were audio-recorded, transcribed verbatim, and analyzed using thematic analysis. Two researchers independently coded the transcripts before discussing and agreeing on a coding framework. Similar codes were grouped into themes and sub-themes through an iterative process. Data triangulation was performed by comparing information obtained from people with disabilities, family members, caregivers, and community leaders to enhance the credibility of the findings. The identified priority needs were then used to co-develop the intervention modules on self-esteem, lifeline, and income-generating activities.

### 2.8 Development, implementation and evaluation of intervention modules

#### 2.8.1 Training

Based on the needs identified during the quantitative and qualitative phases, three intervention modules (Self-esteem, Lifeline, and Management of Income-Generating Activities) were co-developed. The modules were delivered through participatory training sessions using standardized visual materials (picture boxes and illustrated guides) adapted to participants with different literacy levels. Four training sessions were conducted over one week in each country. Each session lasted approximately 2–3 h and combined interactive discussions, practical exercises, role-playing, and participant experience sharing. All training sessions were facilitated by experts in social and inclusive education and in the management of income-generating activities (IGAs). Participants included social workers, members of organizations of people with disabilities, and other project stakeholders. These sessions were designed as a training-of-trainers (ToT) approach, equipping participants with the knowledge, skills, and standardized educational materials required to ensure consistent implementation across both countries. Following the training, these trainers conducted community-based awareness and educational activities targeting community leaders, local authorities, community members, household members, and the close relatives of beneficiaries, thereby promoting wider dissemination and sustainability of the intervention. In addition, social workers from participating health facilities integrated these modules into their routine practice by systematically providing education and awareness sessions for hospitalized patients with disabilities and their caregivers. As a result, the modules have become practical pedagogical tools routinely used by social workers to support psychosocial rehabilitation, social inclusion, and patient empowerment within healthcare settings.

#### 2.8.2 Assessment of outcomes

The pilot evaluation was designed to explore participants’ experiences and perceived benefits following the intervention rather than to measure effectiveness. Consequently, the findings presented below reflect qualitative perceptions of the 12 beneficiaries and should be interpreted as exploratory evidence.

The evolution [in terms of self-esteem, lifeline and management of income-generating activities (IGAs)] of the beneficiaries was analysed from two perspectives: the situation before and after the intervention.

An interview guide and audio recordings were used to assess the social benefits of these modules for the 12 people with disabilities and others. In addition, testimonials collected during the awareness-raising activities reinforced the findings from the interviews.

## 3 Results

### 3.1 Results of sociodemographic, social, and clinical data collection

#### 3.1.1 Participants

The survey included 841 people with disabilities, including 418 (49.7%) in Benin and 423 (50.3%) in Côte d’Ivoire.

#### 3.1.2 Characteristics of people with disabilities

The distribution of people with disabilities according to the different study variables in Benin and Côte d’Ivoire (Table 1) reveals the following results:

**Table 1:** Distribution of people with disabilities according to sociodemographic, economic and clinical variables in Benin and côte d’Ivoire.

Variables	Benin	Côte d'Ivoire	Total
Sex	*n* = 418 (%)	*n* = 423, (%)	*n* = 841, (%)
Female	174 (41.6)	170 (40.2)	344 (40.9)
Male	244 (58.4)	253 (59.8)	497 (59.1)
Type of disability	*n* = 418	*n* = 423	*n* = 841
Motor disabilities	262 (62.7)	292 (69.1)	554 (65.8)
Sensory disability	95 (22.7)	78 (18.4)	173 (20.6)
Cerebral palsy	36 (08.6)	09 (02.1)	45 (05.4)
Multiple disabilities	25 (06.0)	44 (10.4)	69 (08.2)
Etiology of disability	*n* = 418	*n* = 423	*n* = 841
NTD	49 (11.7)	49 (11.6)	98 (11.6)
Other illnesses	243 (58.1)	247 (58.4)	490 (58.3)
Do not know	126 (30.2)	127 (30.0)	253 (30.1)
RSP^a^	*n* = 418	*n* = 423	*n* = 841
Yes	302 (72.2)	252 (59.6)	554 (65.9)
No	116 (27.8)	171 (40.4)	287 (34.1)

a Restrictions on social participation.

The median age was 38 years (Q1 = 22; Q3 = 52), with extreme values ranging from 1 to 115. Three quarters of the respondents were aged between 15 and 65.

Among the people with disabilities surveyed, 497 (59.1%) were men and 344 (40.9%) were women, for a sex ratio of 1.45.

In terms of disability type, 772 people with disabilities (91.8%) had a single disability, whereas 69 (8.2%) had multiple disabilities. Participants with isolated motor disabilities were the largest group (65.8%). A total of 173 people with disabilities (20.6%) had isolated sensory disabilities (visual, auditory, olfactory, or tactile), whereas 45 (5.4%) had isolated cerebral palsy (CP). In addition, 98 people with disabilities (11.7%) had disabilities resulting from neglected tropical diseases.

Two-thirds, or 554 (65.9%), of the people with disabilities surveyed had restrictions on social participation.

#### 3.1.3 Education

The analysis of school attendance was carried out only on people with disabilities aged 5 years or over (school age). Among the 828 respondents, 295 (35.6%) had attended school, whereas 533 (64.4%) had never attended school (Table 2).

**Table 2:** Distribution of people with disabilities according to enrollment in schooling in Benin and côte d’Ivoire.

	Benin (%)	Côte d'Ivoire (%)	Total (%)
Schooling	*n* = 411	*n* = 417	*n* = 828
Yes	94 (22.9)	201 (48.2)	295 (35.6)
No	317 (77.1)	216 (51.8)	533 (64.4)

### 3.2 Factors associated with people with disabilities’ nonenrollment in Benin and côte d’Ivoire

#### 3.2.1 Univariate analysis

In the univariate analysis (Table 3), the results revealed a significant association between non-enrolment and several variables among people with disabilities, namely, type of disability (*p* < 0.001), restrictions on social participation (*p* < 0.001) and country of origin (*p* < 0.001). Indeed, people with disabilities with a disability other than motor disability experience restrictions in social participation, and people with disabilities living in Benin have a greater risk of being unschooled.

**Table 3:** Association between schooling and sociodemographic, economic and clinical variables in Benin and côte d’Ivoire (univariate analysis).

Variables	Schooling		OR	IC95%	*P* value
	No	Yes			
Sex	*n* = 533	*n* = 295			0.102
Male	305 (62.1)	186 (37,9)	1	1	
Female	228 (67.7)	109 (32,3)	1.28	[0.95–1.70]	
Type of disability	*n* = 533	*n* = 295			< 0.001
Motor disability	328 (59.6)	222 (40,4)	1	1	
Sensory	120 (69.8)	52 (30,2)	1.56	[1.08–2.26]	
Cerebral palsy	30 (76.9)	09 (23,1)	2.26	[1.05–4.84]	
Multiple disabilities	55 (82.1)	12 (17,9)	3.10	[1.62–5.93]	
Etiology of disability	*n* = 471	*n* = 231			0.954
Other causes	405 (67.1)	199 (32,9)	1	1	
NTD	66 (67.3)	32 (32,7)	1.01	[0.64–1.60]	
RSP^a^	*n* = 533	*n* = 295			< 0.001
No	157 (55.5)	126 (44,5)	1	1	
Yes	376 (69.0)	169 (31,0)	1.79	[1.32–2.40]	
Country of origin	*n* = 533	*n* = 295			< 0.001
Côte d’Ivoire	216 (51.8)	201 (48.2)	1	1	
Benin	317 (77.1)	94 (22.9)	3.14	[2.32–4.24]	

a Restrictions on social participation.

#### 3.2.2 Multivariate analysis

Multivariable analyses revealed the following (Table 4):

- Participants with multiple disabilities had a 4-fold greater risk of not attending school [ORa = 3.91; 95% CI (2.01–7.2); *p* < 0.001]. In addition, sensory impairment increased the risk of not attending school by 47%.
- Restrictions on social participation also increased the risk of not attending school by 63% [ORa = 1.63; 95% CI (1.19–2.23); *p* = 0.002].
- Finally, people with disabilities living in Benin were three times more likely to be out of school [ORa = 3.10; 95% CI (2.28–4.23); *p* < 0.001]. In the multivariate analysis, three factors were significantly associated with people with disabilities not attending school: type of disability (*p* < 0.001), restrictions on social participation (*p* = 0.002) and country of origin (*p* < 0.001) (Table 4).

**Table 4:** Association between schooling and sociodemographic, economic and clinical variables in Benin and côte d’Ivoire (multivariate analysis).

Variables	Schooling		ORa	IC95%	*P* value
	No	Yes			
Type of disability	*n* = 533	*n* = 295			<0,001
Motor disability	328 (59.6)	222 (40.4)	1	1	
Sensory	120 (69.8)	52 (30.2)	1.47	[1.00–2.16]	0,048
Cerebral palsy	30 (76.9)	09 (23.1)	1.38	[0.62–3.06]	0,426
Multiple disabilities	55 (82.1)	12 (17.9)	3.91	[2.01–7.2]	**<0,001**
RSP^a^	*n* = 533	*n* = 295			0.002
No	157 (55.5)	126 (44.5)	1	1	
Yes	376 (69.0)	169 (31.0)	1.63	[1.19–2.23]	**0.002**
Country of origin	*n* = 533	*n* = 295			<0,001
Côte d’Ivoire	216 (51.8)	201 (48.2)	1	1	
Benin	317 (77.1)	94 (22.9)	3.10	[2.28–4.23]	**<0,001**

a Restrictions on social participation.

Bold values represent statistically significant *p*-values (*p* < 0.05).

### 3.3 Development and pilot implementation of intervention modules

Three key modules have been developed in the form of picture boxes and visual aids adapted to different age groups and literacy levels:

- ***Self-esteem:*** This is an essential module for helping affected individuals restore their inner confidence, their sense of personal worth, and their ability to establish fulfilling relationships with others.
- ***The Lifeline:*** a powerful psychosocial tool designed for people experiencing trauma or psychological suffering. It enables them to become aware of their journeys, recognize their inner resources, and give new meaning to their life experience.
- ***Managing IGAs:*** This module trains participants to plan, manage and organize sustainable economic activities while developing soft skills (leadership, communication, responsibility). (Figure 2).

**Figure 2:**
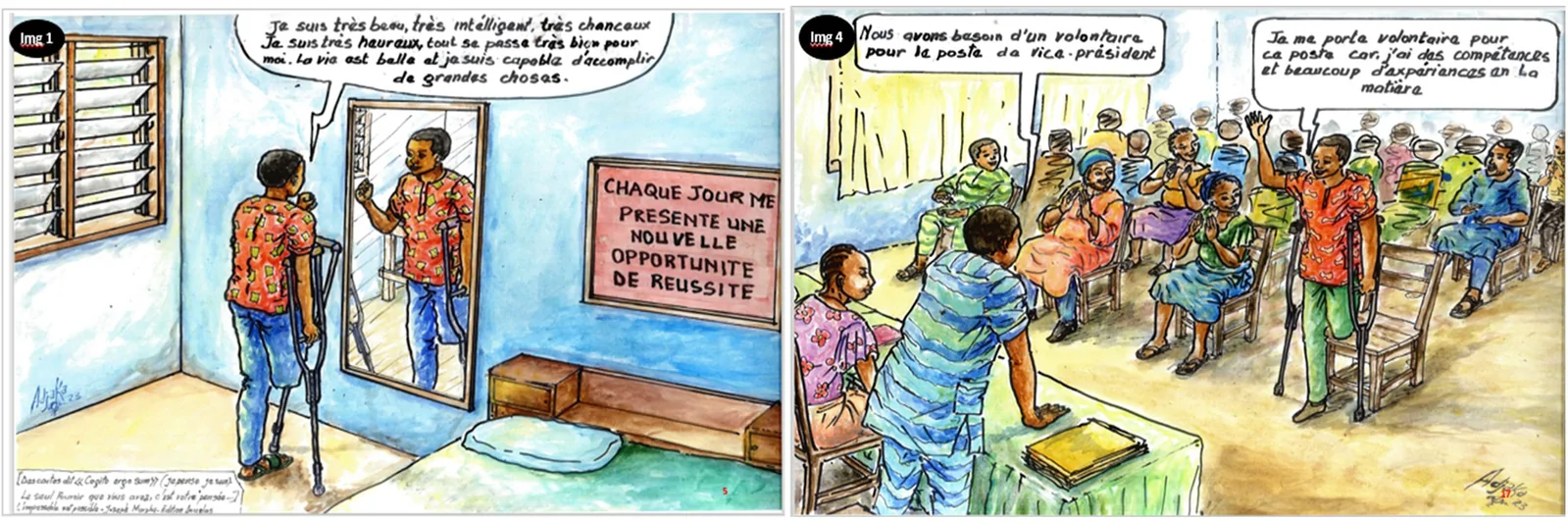
Examples of the illustrated intervention modules (self-esteem).

Prior to the adoption of the pedagogical tools, thematic illustrations of the three modules were presented to and validated by members of the NTD's people with disabilities associations in Benin and Côte d’Ivoire. These modules were then implemented by members of the people with disabilities associations and trained social workers, who then conducted targeted awareness-raising sessions. This approach was perceived by participants as strengthening local capacities and as contributing to positive social change, notably by reducing the isolation of those affected, enhancing their skills, and supporting their inclusion in the community. The tools developed can be viewed on the website https://irfp-benin.com/recherches-and-actions.

Through the use of these tools, participants reported perceived changes within their communities. Several people with disabilities described having learned to make craft objects, liquid soaps, and pottery, which they felt contributed to their financial independence and professional integration.

Below are examples of how this program has transformed or improved the lives of people with disabilities.

#### 3.3.1 From social and community education to perceived social transformation

In contexts where people affected by NTDs experience multiple forms of stigmatization, social and community education is a strategic lever for transforming perceptions, rehabilitating their social status and restoring their dignity.

Indeed, most of the discrimination experienced by these people is based on ignorance, erroneous beliefs or devaluing social representations. In many communities, visible disabilities linked to NTDs, such as leprosy or Buruli ulcers, are interpreted through magico-religious reading grids, leading to rejection, fear and pity.

Participants reported that social education helped to challenge these representations and provided spaces for dialogue, awareness-raising and collective learning, adapted to the local cultural context. The discussions were punctuated by a number of testimonials from participants, some of which were selected for illustrative purposes:

“When my son introduced me to his girlfriend, who has a disability, I initially refused to accept her. After participating in the awareness session and listening to the testimonies, I realized how unfair my attitude had been. Today, I understand that people with disabilities deserve the same respect and opportunities as everyone else.” (Parent, Adja-Ouèrè).

During one of the community awareness-raising sessions, a young man spoke up, saying, “For many years, I was deeply ashamed of my disability. I avoided wearing clothes that would reveal my affected foot because I feared being mocked and rejected by others. During the self-esteem sessions, I met other people with disabilities who openly accepted themselves and shared their experiences without fear. Their testimonies, together with the community awareness activities, gave me the confidence to change the way I saw myself. Today, I no longer feel the need to hide my disability. I can openly show who I am, and I have learned to accept myself with dignity” (spontaneously, he receives encouragement and applause from the participants). (a young disabled participant).

These interventions, led by people with disabilities themselves, were perceived by participants as helping to humanize the way affected people are viewed by emphasizing their value, rights and abilities; promoting their social reintegration; deconstructing the mental and symbolic barriers that isolate them; and informing communities about the causes, modes of transmission and treatment options for NTDs.

In addition, community education encourages the active participation of opinion leaders, community health workers, teachers, religious leaders and local authorities, as shown by the testimonies of a few opinion leaders.

“I am the village chief, and I also live with a disability. My disability has never prevented me from serving my community. Leadership depends on the mind and the heart, not on physical appearance.” (village chief, Lalo district).

The participation and testimony of these figures play a central role in disseminating empowering messages and legitimizing respectful behavior. Furthermore, by training members of the disabled and affected people's associations as trainers in the use of tools related to self-esteem, lifelines, and IGAs by giving them the floor for testimonials during community sessions, workshops and forums, these educational schemes appeared to reinforce their self-esteem, which is a condition for them to be able to claim their dignity and be respected.

“Through the self-esteem and lifeline training, I realized that my story could inspire others. Today, I train other people with disabilities and speak confidently during community sessions. I have regained my dignity and my voice.” [Ms. B, member of the “Association des Personnes Handicapées et Affectées de la lèpre, l’ulcère de Buruli et autres Maladies Tropicales Négligées à manifestation cutanée (APHA-MTNc)” Benin board].

In short, social and community education is a prerequisite for any psychosocial rehabilitation process, as it creates an environment conducive to a change in outlook, living together and mutual recognition. Without this component, medical or economic efforts risk remaining incomplete or even ineffective in the long term.

#### 3.3.2 From rejection to respect and restored dignity

The testimonies gathered from people with disabilities affected by neglected tropical diseases, i.e., beneficiaries of the “Disability Today” project, vividly illustrate the path they have taken from stigmatization to recognition. Their words, rooted in their own lives, reveal the extent to which social education initiatives, opportunities for self-expression and the valorization of life skills have contributed to restoring not only their self-esteem but also their place in society. There are a few significant extracts from these transformative stories.

Mrs D: “Before this project, I was rejected by my family and community because of my disability. Many people considered me incapable, and I was discouraged from pursuing the career I had chosen. After completing my vocational training through the project and receiving support to start my own business, my life changed considerably. Today, I work alongside my colleagues, my family and community respect me, and I am recognized for my abilities rather than my disability. The community awareness activities also helped change people's attitudes toward me. I have gone from being rejected to being admired, respected, and even considered a role model for others.” (Mrs D, beneficiary and member of APHA-MTNc).

Mrs. L: “Before joining the project, I was ashamed to leave my home. After receiving vocational training in hairdressing, IGA, and participating in community awareness activities, I became more confident, socially accepted, and actively involved in my community. Today, I’m accepted, and I have a voice, despite my refusal to undergo surgery. I’m more than ever a committed figure in my community and even in politics (smile).” (Mrs L, beneficiary, Lalo).

Mr. J: “The project transformed my life. I now have a better living environment, greater self-confidence, and feel respected within my community. I’m not a leader yet, but it is as if I already am (laughs).” (Mr J, beneficiary, Lalo).

These testimonies are apt reminders that dignity and respect cannot be decreed but rather are built through experience, recognition and a newfound voice. By giving those affected the opportunity to act, train and bear witness, educational interventions repair much more than prejudice: they repair bonds, identities and hope. Every voice heard becomes a lever for change in every community.

Restoring dignity and respect for people living with disabilities related to neglected tropical diseases is not just a moral or humanitarian imperative; it is an essential condition for any equitable, sustainable and human rights-based public health approach.

Beyond medical care and access to schooling, it is essential to act on the social and symbolic dimensions of exclusion. From this perspective, social and community education emerges as a fundamental lever. By deconstructing stereotypes, rehabilitating the value of those affected and encouraging their active reintegration into community life, it contributes directly to recreating the conditions for respectful, supportive and inclusive living together.

This calls for a comprehensive approach that combines individual support, collective transformation and institutional commitment. It must aim not only to relieve physical suffering but also to restore the moral, social and identity integrity of those affected. A truly inclusive society can be recognized for its ability to protect the dignity of each individual and make mutual respect a collective norm.

## 4 Discussion

The data collected during our study enabled us to identify the factors associated with the nonenrollment of people with disabilities in Benin and Côte d’Ivoire, including the type of disability, restrictions on social participation and country of origin.

### 4.1 Country of origin

There is a difference according to the country of residence of people with disabilities. People with disabilities living in Benin are 3 times more likely to be out of school than those living in Côte d’Ivoire. This difference may be due to political and administrative differences between the two countries (11, 12).

On the other hand, these observations can be explained by the difference in levels of development between the two countries. Indeed, even though both countries are classified as middle-income countries (lower-middle-income for Benin and upper-middle-income for Côte d’Ivoire) (13, 14), Côte d’Ivoire's gross domestic product (GDP) is much higher than that of Benin ($2500 versus $1,300 in 2023) (15, 16). The more developed a country is, the more opportunities it offers its inhabitants.

### 4.2 Types of disability

The type of disability was also associated with non-schooling among people with disabilities, regardless of sensory disability (47%), cerebral palsy (38%) or multiple disabilities (4 or more). In this context, inclusive education can be a solution. Anne-Laure Perrin et al., in France in 2022, reported that public attitudes toward inclusive education differ according to the type of disability presented. More specifically, attitudes are less positive toward students with intellectual disabilities such as Down's syndrome or autism spectrum disorder than toward those with motor or sensory disabilities (17). Motor and sensory disabilities seem to be more tolerated. Indeed, according to Fatoye et al., most of the time, perceptions of disability determine the way people treat or interact with people with disabilities. In addition, a large proportion of the population has a negative attitude toward people with disabilities on the basis of a lack of awareness of their needs (18).

Danielle Tan noted that in West African countries such as Mali, Niger and Burkina Faso, representations of disability are mainly spiritual, divine and relational. Disability is most often seen as a “tragedy”, or a “punishment” inflicted on the family for cursing evil spirits, leading to discrimination and shame for all members (19). In Ethiopia, Adugna et al. reported that children with physical disabilities in low- and middle-income settings frequently experience stigma across multiple environments, including the home, community, and school. This stigma manifests through bullying, pity, social exclusion, and discrimination, with adverse effects on their emotional well-being, social relationships, and educational participation (20). These findings highlight the need for comprehensive and context-specific interventions, including awareness-raising initiatives, empowerment strategies, and the strengthening of inclusive education policies and practices to reduce stigma and improve outcomes for these children.

### 4.3 Social participation

Social participation restrictions constitute an essential dimension of disability, as defined by the World Health Organization's International Classification of Functioning, Disability and Health (ICF). This refers to the limitations faced by people with disabilities in their participation in activities of daily living, particularly education. Furthermore, the lack of measures taken to facilitate social participation can be a key factor in the enrollment rate (21). Indeed, Amani Bettioui noted the inaccessibility of roads leading to schools, the inaccessibility of school space, financial problems for parents of pupils with disabilities, financial difficulties for stakeholders such as people with disabilities organizations, and educational offerings that are highly unsuited to the educational needs of children with disabilities, which manifest themselves at several levels, namely, curricula, teaching aids, teaching methods (22) and assessments. N’chot Julie, 2022, in Côte d’Ivoire also noted the restrictive school infrastructure for disabled students at the University Félix Houphouet-Boigny Abidjan (23).

### 4.4 Interventions

In addition to the quantitative aspects raised by these results, modules have been developed to meet the needs of people with disabilities. These activities have improved self-esteem, provided a lifeline and facilitated access to and management of income-generating activities for people with disabilities. A study by Lestari et al. revealed that social support among people with physical disabilities contributed to 47% of their self-esteem. These authors reported that people with physical disabilities had average social support and average self-esteem. Research suggests that social rehabilitation centers optimize social support in the form of emotional support, information, tools and recognition, as this plays a role in the development of self-esteem among people with physical disabilities (24). One of the goals of education (schooling and income-generating activities) is to enable individuals to meet their basic needs in the short or long term. Income-generating activities and livelihood programs significantly strengthen women's financial independence, improve their ability to influence decisions within the household and the community, and enhance their social status (25, 26). Participants’ testimonies suggest that the intervention contributed to improvements in self-esteem, social participation, and engagement in income-generating activities. However, these findings are based on qualitative perceptions from a small pilot sample and should be interpreted as preliminary evidence. Future studies using larger samples and quantitative outcome measures are needed to formally evaluate the effectiveness of these intervention modules.

This study has several limitations. It was not possible to include all known aspects of education. Additionally, not all findings from Benin and Côte d’Ivoire can be transmitted to all countries.

## 5 Conclusion

This study identified factors associated with the non-schooling of people with disabilities in Benin and Côte d’Ivoire, including restrictions on social participation, country of origin, and type of disability. It also supported the development of tools to strengthen self-esteem, life planning, and income-generating activities.

These findings show that educational inclusion goes beyond school attendance and is shaped by broader social, cultural, and economic factors. They highlight the need to adapt school systems, strengthen inclusive education programs, and provide psychosocial and financial support to families and communities.

Overall, this study emphasizes the importance of context-specific strategies to improve access to education and promote the social inclusion of people with disabilities in low- and middle-income countries.

## Data Availability

The datasets presented in this study can be found in online repositories. The names of the repository/repositories and accession number(s) can be found in the article/Supplementary Material.
